# The Neurobiology and Treatment of Restless Legs Syndrome

**DOI:** 10.3233/BEN-2012-120271

**Published:** 2013

**Authors:** Ruth Jones, Andrea Eugenio Cavanna

**Affiliations:** ^1^The Michael Trimble Neuropsychiatry Research GroupDepartment of NeuropsychiatryBSMHFT and University of BirminghamBirminghamUK; ^2^Department of Motor Neuroscience and Movement DisordersInstitute of Neurology and University College LondonLondonUK

**Keywords:** Dopamine, iron, opioids, pathophysiology, pharmacotherapy, restless legs syndrome

## Abstract

*Background:* Restless legs syndrome (RLS) is a relatively common neurological disorder affecting sleep and health-related quality of life. Neuroimaging studies, autopsy investigations and experimental studies using animal models have been conducted to investigate the potential causes of RLS, resulting in the generation of multiple pathophysiological hypotheses.

*Methods:* This paper reviews the neurobiology and pharmacotherapy of RLS, with a critical analysis of the heterogeneity and methodological limitations of the existing scientific literature.

*Results:* Although several neurotransmitter systems dysfunction and neuroanatomical abnormalities have been implicated in RLS pathogenesis, dopamine dysfunction within basal ganglia pathways, iron deficiency and opioid system abnormalities have consistently been found to be involved. Their involvement is further strengthened by the therapeutic effectiveness of dopaminergic agents, iron supplementation and opioid medications.

*Discussion:* Converging evidence from neuroimaging, autoptic and animal studies points towards dopamine dysregulation and iron metabolism alterations as the main contributors to RLS pathophysiology. The possible interactions between different neurotransmitter systems should guide further neuropharmacological research in order to improve therapeutic efficacy for this disabling condition.

## 1. Introduction

Restless Legs Syndrome (RLS) is a chronic sleep-associated neurological disorder bearing a significant impact on sleep quality, daytime functioning and health-related quality of life. RSL is relatively common, affecting 2.5 to 15% of the US general population, with prevalence rates increasing alongside age [1]. Around 2–3% of patients experience significant symptoms severity, requiring long-term pharmacotherapy [2]. RLS is identified by leg uneasiness during immobility, causing an urge to mobilize the legs to overcome this sensation. Symptoms generally peak during the evening, and arms can (less often) be involved. Most patients affected by RLS experience Periodic Leg Movements during Sleep (PLMS) or wakefulness (PLMW), although this is not an essential diagnostic criterion [3]. The diagnosis of RLS is clinical, and the differential diagnoses with conditions resembling RLS are shown in Table 1.

The underlying pathophysiology of RLS is not fully understood, a factor which is likely to hinder successful management. However, different pharmacological therapies are routinely used to treat RLS, such as dopaminergic agents (both L-dopa and dopamine agonists), opioids, benzodiazepines and anticonvulsants (e.g. gabapentin and its pro-drug gabapentin enacarbil). DA agonists remain the first-line therapy despite the frequent phenomena of augmentation and rebound,

ISSN 0953-4180/13/$27.50 © 2013 – IOS Press and the authors. All rights reserved

Table 1
Differential diagnosis of restless legs syndrome

| Peripheral neuropathies | Akathisias | Peripheral vascular disease | Leg cramps at night | Painful legs along with moving toes |
|---|---|---|---|---|
| – Circadian alterations absent<br>– PLMS absent<br>– Dysfunctional nerve conduction<br>– No relief from moving | – Circadian alterations absent<br>– Paraesthesia absent<br>– DA antagonists relieve symptoms | – Exacerbation from moving, improvement from resting<br>– Vascular along with skin alterations | – Unilateral<br>– Localized<br>– Rapid, intense/serious onset | – Desire to move absent<br>– Exacerbation when resting absent<br>– Relief from moving absent<br>– Circadian alterations absent |

Abbreviations: PLMS, Periodic leg movements during sleep; DA, Dopamine.

which cause symptoms to become more severe and begin earlier during the day [2]. Side-effects including nausea, fatigue, somnolence and dizziness are also frequently reported.

Numerous hypotheses have been proposed to explain the underlying pathophysiology of RLS. These include genetic variations, peripheral neuropathy, spinal cord microlesions, brain metabolism fluctuations, iron deficiency and neurotransmitter dysfunctions. RLS is currently viewed as a network dysfunction encompassing different areas involved in somatosensory perception alongside motor production [4]. This article reviews the literature on the neuobiological underpinnings and available pharmacological therapies of RLS. Although both variants of RLS (primary and secondary) will be covered, the main focus will be upon primary RLS and its pathophysiology, in an attempt to identify the most effective treatments available for this common condition.

### *1.1. Primary and Secondary RLS*

Primary (idiopathic) RLS has a strong genetic component and is characterized by a younger age of onset compared to secondary RLS [4]. Approximately 60% of sufferers have a positive family history and genetic research has identified five genes along with ten potential alleles associated with RLS. A specific difference in one allele has been related to reduced serum ferritin, implying an overall decrease in bodily iron storage [5]. Genetically-driven dysfunction within central dopamine systems and iron metabolism seem to be the main factor involved in the pathophysiology of RLS [6].

Three possible causes have been proposed for secondary RLS: End-Stage Renal Disease (ESRD), iron deficiency and pregnancy. The key role of iron deficiency has been supported by research findings investigating serum along with cerebrospinal fluid (CSF) iron levels, neuroimaging studies and clinical studies focusing on treatment response to IV iron [7].

## 2. Dopamine (DA) pathophysiology model

Since initial reports on effectiveness in RLS [8], levodopa (L-dopa) has been continuously used along with DA agonists as an efficacious treatment option for RLS and PLMS [9]. The substantial effectiveness of dopaminergic pharmacotherapy (above 90% using L-dopa) is consistent with the hypothesis that dysfunction within DA systems generates RLS symptoms [10]. A possible pathophysiological model suggests that RLS is a hyper-dopaminergic disorder resulting in postsynaptic desensitization and circadian reduction of DA activity at night. Unfortunately pharmacological dopaminergic stimulation frequently leads to augmentation: long-term DA agonist pharmacotherapy can consequently produce more severe symptoms. In these cases, non-dopaminergic alternatives could prove useful [5]. The established effectiveness of dopaminergic medications is consistent with a central role for the nigrostriatal pathway, which regulates voluntary motor activity, in the pathophysiology of RLS [11].

### *2.1. Imaging investigations*

A number of neuroimaging techniques, including functional magnetic resonance imaging (fMRI), positron emission tomography (PET) and single photon emission computer tomography (SPECT), have been used to investigate dopaminergic neurotransmission abnormalities in RLS. Neuroimaging studies have often generated inconsistent findings (Table 2) [12]. PET and SPECT studies have yielded variable results in terms of pre- and post-synaptic DA neurotransmission and it is not clear exactly how alterations in brain metabolism

Table 2
Neuroimaging studies on the role of dopamine in the pathophysiology of restless legs syndrome

| Study | Year | Methods | Patients | Results | Main limitations |
|---|---|---|---|---|---|
| Staedt et al. [17] | 1995 | Ligand selective for DA D2 receptors, [$^{123}$I]IBZM and SPECT explored DA D2 receptor binding. | 20 NMS sufferers compared to healthy controls. | NMS sufferers demonstrated reduced striatal [$^{123}$I]IBZM binding. Reduced DA D2 receptor binding associated with NMS. | Controls represented an early-onset age of RLS. NMS sufferers were older. Not all NMS sufferers experienced RLS. Binding could have been affected by changes in receptor concentration, sensitivity, and competition *in-vivo* between endogenous or exogenous ligands and radioligand. |
| Trenkwalder et al. [13] | 1999 | [F18] flurdeoxyglucose (FDG) and (F18) flurodopa [$^{18}$F]dopa PET. | 6 RLS sufferers, 4 patients and healthy age-matched controls. | Regional blood flow (shown by FDG) did not significantly differ between groups. [$^{18}$F]dopa demonstrated no binding differences. Normal overall and area specific glucose metabolism for RLS sufferers. | PET unable to identify resting brain metabolism dysfunction or presynaptic DA abnormalities under identifiable threshold. Possible abnormalities in tissue unavailable to scanner detection. Functional abnormalities would remain undetected in asymptomatic patients. |
| Turjanski et al. [14] | 1999 | [$^{18}$F]dopa investigated storage of terminal DA within nigrostriatal pathways. [$^{11}$C]raclopride PET explored striatal D2 receptor binding. | 13 RLS sufferers alongside age-matched controls. | RLS sufferers demonstrated lower average D2 receptor binding throughout caudate and putamen. 6 patients showed decreased binding in both caudate and putamen, 3 only in the putamen. Patients receiving L-dopa did not differ in 18F-dopa uptake or striatal D2 receptor binding. | Reduced binding could result from endogenous DA abnormalities, downregulation or greater occupancy of receptors. |
| Ruotinnen et al. [15] | 2000 | [$^{18}$F]dopa with PET explored presynaptic DA functioning within nigrostriatal pathways. | 9 untreated RLS sufferers with PLMS compared to 27 controls. | Small but significant reduction in striatal [$^{18}$F]dopa uptake among patients for both caudate and putamen. | Possible differences in DA regulation between individuals who only suffer RLS and those who additionally experience PLMD. Focused on the nigrostriatal pathway only. Identifying altered pathways can be problematic due to resolution difficulties of PET and sensitivity issues with [$^{18}$F]dopa. |
| Eisensehr et al. [19] | 2001 | $^{123}$I-IPT SPECT and [$^{123}$I]IBZM SPECT investigated striatal presynaptic DA transporter concentration alongside postsynaptic DA receptor density. | RLS sufferers (14 untreated and 11 on L-dopa) compared to 10 controls. | Patients and controls showed comparable binding to striatal DA transporters and D2 receptors. D2 receptor binding reduced with ageing. | Age differences between treated (elderly) and untreated patients (midaged). |
| Michaud et al. [18] | 2002 | [$^{123}$I]b-CIT and [$^{123}$I]IBZM SPECT investigated striatal presynaptic and postsynaptic DA binding. | 10 untreated patients with RLS and PLMS compared to 10 controls. | Patients did not differ in [$^{123}$I]b-CIT binding compared to controls. Patients demonstrated significant reductions for striatal D2 receptor IBZM binding. | PET and SPECT do not differentiate between reduced receptor quantity and lower radioligand affinity. Other dopaminergic pathways possibly involved were not investigated. RLS with PLMS can be individually or collectively related to D2 receptor binding abnormalities. |

Table 2, continued

| Study | Year | Methods | Patients | Results | Main limitations |
|---|---|---|---|---|---|
| Tribl et al. [20] | 2002 | [$^{123}$I]IBZM SPECT explored DA receptors. | 14 RLS sufferers compared to gender and age-matched controls. | – No significant differences in postsynaptic concentration of striatal DA D2 receptors.<br>– No significant differences between patients receiving DA agonists, other pharmacotherapy or no treatment. | |
| Červenka et al. [16] | 2006 | PET [$^{11}$C]raclopride along with [$^{11}$C]FLB 457 explored available striatal and extrastriatal DA D2 receptors. | 16 untreated patients with RLS and 16 controls. | [$^{11}$C]raclopride striatal binding capability significantly greater for RLS patients.<br>[$^{11}$C]FLB 457 extrastriatal binding significantly increased for patients (esp. thalamus and anterior cingulate cortex). | |

Abbreviations: [123I] labeled (S)-2-hydroxy-3-iodo-6-methoxy-([1-ethyl-2-pyrrolidinyl]methyl) benzamide ([$^{123}$I]IBZM); Single Photon Emission Computer Tomography (SPECT); Dopamine (DA); Nocturnal Myoclonus Syndrome (NMS); Restless Legs Syndrome (RLS); [F18] flurdeoxyglucose (FDG); 6-[18F]fluoro-l-dopa ([$^{18}$F]dopa); positron emission tomography (PET); Periodic Limb Movements during sleep (PLMS); ($^{123}$I)-(N)-(3-iodopropen-2-yl)-2$\beta$-carbomethoxy-3$\beta$-(4-chlorophenyl) tropane ($^{123}$I-IPT).

reflects in the central DA pathways abnormalities observed in RLS [12].

Studies using PET have yielded inconsistent findings with regards to brain DA receptor binding. One study [13] demonstrated no binding differences, two studies found minor decreases in striatal receptor binding [14,15] and another study [16] reported increased striatal and extrastriatal binding capability. Studies using SPECT to investigate striatal receptor binding are also conflicting. Two studies [17,18] reported reduced binding for patients compared to controls, while another study [19] found no differences. No differences were observed using [$^{123}$I]b-CIT [18]. In one study [20] striatal DA receptor concentration was comparable between patients and controls.

Converging evidence from different studies suggests dysfunctioning DA pathways [14,21]. Lower uptake in the putamen and striatum was not likely to reflect neuronal loss. However, there is some evidence suggesting minor hypofunctioning of nigrostrial DA presynaptic systems in patients with RLS and PLMD [15]. Normal functioning of D2 receptors as well as DA transporters throughout the striatum has been implicated [19].

This central DA abnormality hypothesis in RLS with PLMS is consistent with imaging investigations demonstrating striatal D2 receptor dysfunction [18]. There are a few possible explanations for the reduced binding to D2 receptors. Both D2 auto-receptor abnormalities and reduced enzyme activity for the metabolism of DA (monoamine oxidase/catechol-o-methyltransferase) could result in increased amounts of endogenous DA within the synaptic cleft. Increased DA concentrations can result in receptor downregulation and a reduction in D2 receptor number or IBZM affinity [18]. The increased synaptic levels of DA can result in competition for D2 receptors amongst endogenous DA and the radioligand, with dislodgment of [$^{123}$I]IBZM from receptors.

One study [17] observed DA dysfunction amongst patients with NMS. DA agonists are an effective treatment for both RLS and Nocturnal Myoclonus Syndrome (NMS), a condition characterised by stereotypical repeated jerk-like movements of lower limbs whilst awake or asleep and frequently associated with RLS.

Although imaging studies have particularly investigated nigrostriatal structures, alternative extra-nigral DA or non-DA pathways could be affected in RLS [44]. As both PET and SPECT have limited spatial resolution, dopaminergic alterations in extra-striatal areas (sub-striatum, brainstem and spine) would not have been identified [39]. The PET imaging technique reflects the competitive absorption of ligands. This could therefore be affected by membrane receptor concentrations or DA levels within the synapse. Complex interactions between endogenous ligands could also produce inconsistent findings across these imaging studies [25]. The reviewed neuroimaging studies were performed throughout the daytime when RLS symptoms are mild and cannot be observed, or are less noticeable. Imaging conducted during the evening and nighttime when RLS is typically expressed could have identified greater dysfunction [9]. Furthermore, different radioligands can vary in their binding affinities and generate different findings.

### 2.2. Cerebro-spinal fluid (CSF) investigations

One study [22] measured amine metabolites in CSF and blood samples from 22 drug-naive moderate-to-severe RLS sufferers compared to 11 control participants. CSF levels of homovanillic acid (HVA), L-dopa, serotonin (5-HT) and 3-othro-methyl-dopa (3-OMD) did not significantly differ between groups. Likewise, this study reported normal blood 5-HT concentrations in the two groups.

Another investigation [23] explored daily fluctuations in DA function. CSF was obtained from 30 RLS sufferers and 22 control subjects at 10pm and analysed for metabolites associated with DA. These were then compared to previous CSF samples obtained at 10am from a prior investigation. Non-significant fluctuations were observed for control participants. In contrast, patients with RLS demonstrated noticeable daily fluctuations with increases in tetrahydrobiopterin (BH4), 3-OMD, HVA and SH1AA between the 10 am and the 10 pm samples. These differences implicate DA circadian changes which are larger than in the general population. Elevated BH4 levels could indicate a greater requirement of DA in RLS, which could occur at a particular stage of the circadian production of DA.

CSF levels of 3-OMD are often elevated in RLS sufferers, suggesting greater L-dopa production, limited L-dopa decarboxylation or increased MAT/catechol-*O*-methyltransferase (COMT) functioning. One study [24] investigated whether abnormalities in 3-OMD levels in patients with RLS are due to greater DA and HVA production. In this study, CSF HVA was significantly increased in RLS sufferers who had greater 3-OMD levels. Decreased CSF ferritin was also reported in patients with increased 3-OMD levels. Patients with higher 3-OMD levels demonstrated more PLMS, suggesting greater RLS severity possibly mediated by increased DA production.

### 2.3. Post-mortem studies

Post-mortem studies explored brain iron-deficiency alongside dopaminergic abnormalities [25]. The substantia nigra and putamen of 8 patients with young-onset, moderate-to-severe RLS and 15 normal controls were retrieved during autopsy. The patients were characterized by significant reductions in D2 receptors within the putamen compared to controls. RLS was also associated with increased concentration of Tyrosine Hydroxylase (TH) within the substantia nigra only. Significant increases in phosphorylated (active) TH along with TH were seen in the substantia nigra and putamen. DA receptors, transporters and VMAT did not differ for the putamen or substantia nigra. In-vivo animal models of iron deficiency and in-vitro cellular models also demonstrated significant increases in TH and phosphorylated TH, comparable to RLS autopsy findings. This is consistent with the hypothesis that DA dysfunction resulting in hyperactive DA system can be related to iron deficiency. However, it is important to note that since patients with RLS had also received dopaminergic or non-dopaminergic pharmacotherapies prior to the study, these findings might have been confounded.

Another autoptic study explored A11 cell bodies from 6 patients with primary RLS and 6 age-matched controls [26]. Staining for TH along with stereological assessment of the cell volume were performed for each TH positive cell. Gliosis within the area was also investigated. TH positive assessment, fractional GFRP staining, cell volume and standard histological staining did not show any significant differences between the samples. These findings suggest that cell loss and neurodegeneration do not occur within the A11 hypothalamic area in RLS. However this does not rule out completely the possibility of A11 involvement. Brain circuitries other than nigro-striatal DA pathways were not investigated. Moreover, DA metabolic alterations within the cells, distal synaptic changes or abnormalities in receptors and transporters were not assessed and might not be identified by structure differences. In general, autopsy studies were conducted on relatively small samples, resulting in difficulties in identifying accurate neuronal alterations.

Finally, autopsy investigations were only performed in patients with RLS whose symptoms commenced below 45 years of age. Different pathophysiologies have been demonstrated between young and older-onset RLS [9], therefore samples should ideally not be restricted to a particular age range.

### 2.4. Animal studies

The possible involvement of A11 DA diencephalic spinal neurons in RLS has been investigated in an animal model [21]. The A11 nucleus of rats was stereotactically lesioned with bilateral injection of 6-

hydroxydopamine (6-OHDA) to induce behaviours comparable to RLS. Pathological assessment found A11 TH stained cells were decreased by 54% for four 6-OHDA rats compared to two sham rats. Total standing duration was greater for the lesioned rats compared to the sham rats, with no significant differences in overall sleep duration between the two groups. Lesioned rats subsequently given pramipexole demonstrated reduced standing incidence and overall standing duration, which could support the effectiveness of DA agonists in reducing the symptoms of RLS.

A possible limitation of this study is that RLS in rats may manifest differently or be caused by different pathophysiological mechanisms compared to humans and other primates. For example, movements similar to PLMS were not observed. Moreover, monitoring was irregular, electrophysiological assessment of sleep was not conducted and subjective consequences of RLS could not be established. The small sample size also limits the generalisability of these findings.

## 3. Iron pathophysiology model

Iron deficiency has been consistently associated with RLS. Numerous investigations have been performed exploring CSF alongside serum iron, ferritin and transferrin concentrations. The hypothesis of iron involvement is further strengthened by the therapeutic efficacy of both oral and IV iron supplementation for patients with RLS. Reduced iron concentration within the substantia nigra of RLS sufferers has been demonstrated with MRI and autopsy. Decreased ferritin and iron in CSF of patients presenting with normal serum iron levels have also been reported. Specifically, insufficiency of brain iron storage has been demonstrated to be more severe for young-onset RLS than older-onset [10]. DA activity is affected by iron insufficiency, through the increase of TH levels, consequently heightening extracellular DA: this results in decreased dopamine transporter (DAT) expression on cell surfaces and a marked reduction in D2 receptors for severely affected patients [5]. However the precise process causing iron-deficiency is not fully understood [27].

An early study investigated the short-term effect of iron-deficient anaemia on rat brain monoamine processing using in-vivo microdialysis [28]. Significant increases in DA and its metabolites along with DA were demonstrated amongst iron-deprived rats fed in the dark in comparison to controls, which remained unaltered. These findings implicate that monoamine absorption and metabolism is altered in experimental models of iron insufficiency.

Another study [29] assessed CSF and serum iron from 16 patients with idiopathic RLS and 8 age-matched healthy individuals. CSF ferritin concentrations were reduced and transferrin increased in RLS, whilst serum ferritin and transferrin concentrations did not differ between patients and controls.

In a neuropathological study on 7 patients with primary RLS compared to 5 age-matched controls with no neurological abnormalities, no anomalies were detected for iron staining in the substantia nigra and for immunohistochemistry testing of TH [30]. However within the RLS substantia nigra, iron and H-ferritin staining was noticeably reduced whereas staining of L–ferritin was robust. Moreover, RLS samples demonstrated reduced transferrin receptors in stained neuromelanin cells, suggesting that abnormalities in obtaining iron within neuromelanin cells and transferrin receptor control deficiency could be involved in the pathophysiology of RLS. Overall deficient iron within the substantia nigra could damage DA metabolism, restricting TH functioning, affecting DA receptor or transporter expression. Thus, impaired iron uptake within neuromelanin cells can eventually result in DA dysfunction.

Neuromelanin cells from the substantia nigra of 4 patients with primary RLS and 4 controls were investigated to assess iron control protein mechanisms [31]. RLS neuromelanin cells showed reduced ferritin, ferroportin, divalent metal transporter 1 and transferrin receptors, whilst transferrin concentration was increased compared to controls. Iron control protein 1 (IRP1) density within neuromelanin cells along with IRP1 quantity was reduced in RLS, whereas IRP2 was increased. These findings are suggestive of an underlying brain iron deficiency where reduced IRP1 produces transferrin receptor mRNA destabilization.

Overall, this study provides further evidence that iron insufficiency within neuromelanin cells could be responsible for DA abnormalities in RLS.

Another study explored the association between Thy-1 (a molecule responsible for cell adhesion and neurotransmitter release from vesicles) and iron in RLS [32]. Autoptic specimens of substantia nigra were obtained from 4 patients with primary RLS and 4 healthy controls. Pheochromocytoma cells (PC12) were investigated for a direct association between cell iron and Thy-1 expression. A significant reduction in Thy-1 expression within PC12 cells occurred during iron chelation, suggesting an association between iron insufficiency and Thy-1 expression at the level of the substantia nigra in RLS. The authors suggested that Thy-1 within neurons can be affected by iron and reduced Thy-1 could be responsible for effects of iron-insufficiency on DA functioning, as Thy-1 expression occurs prior to dopaminergic neurons terminal development, possibly regulating axonal growth and synaptic connectivity.

Several studies have confirmed iron deficiency as an important pathophysiological factor in RLS. A biochemical study on 10 patients with idiopathic RLS found reduced CSF iron and ferritin concentrations, along with increased transferring, compared to 10 age-matched patients with psychophysiological insomnia not experiencing RLS symptomatology [33]. The echogenicity of the substantia nigra was determined in 20 patients with primary RLS, and found to be lower than in 20 patients with idiopathic PD, but higher than in 20 age-matched healthy controls [34]. As tissue iron levels affect the echogenicity of the substantia nigra, this finding suggests a deficit nigral iron in RLS. Finally, a MRI study found significant reductions in substantia nigra iron concentrations for 22 young-onset RLS patients in comparison to 39 healthy controls, but no differences were observed between 19 patients with older-onset RLS and controls, suggesting that brain iron deficiency might be specific to young-onset RLS sufferers [35].

## 4. Opioid system pathophysiology model

The opioid neurotransmitter system has also been implicated in the pathophysiology of RLS due to the treatment effectiveness of opiates, specifically for patients manifesting painful symptoms. Furthermore, the administration of naloxone (an opiate antagonist) to patients with RLS taking opiates causes symptoms to reoccur [36,37]. The therapeutic effect is thought to be mediated by the opioid neurotransmitter systems, including endorphins and enkephalins [38].

A study investigated the levels of Beta-endorphin, Met-enkephalin and Leu-enkephalin within the substantia nigra and thalamus of 5 patients with RLS in comparison to 5 controls [38]. The patients with RLS demonstrated decreased Beta-endorphins and Met-enkephalin positive cells within the thalamus compared to controls. Thalamic Leu-enkephalin and nigral Beta-endorphin, Met-enkephalin and TH did not differ between groups. The authors suggested that these findings implicate changes within the central processing of pain. Mu opiate receptors might particularly be involved, as Beta-endorphin and Met-enkephalin (but not Leu-enkephalin) are selective to this receptor type. However alternative opioid regions (red and raphe nuclei) have been suggested to be involved.

A PET study explored the availability of opioid receptors using the non-specific opiate receptor radioligand ($^{11}C$) diprenorphine in 15 patients with idiopathic RLS outpatients from a specialized clinic and 12 age-matched controls [39]. Patients were either not medicated or on low doses of L-dopa or DA agonists, which were discontinued before PET imaging. There were no differences in opioid receptor binding between RLS patients and healthy subjects, however in regions involved in pain processing (medial thalami, caudate, amygdalae, anterior angulate gyri, insular cortices and orbitofrontal cortices) there was a negative correlation between ligand binding and RLS severity. At the level of the orbitofrontal cortices and anterior cingulate gyri, ratings of pain perception demonstrated inverse correlations with opiate receptors density. This suggests that greater severity of RLS symptoms can result in increased endogenous opiate release in brain regions involved in pain processing, which can possibly explain their involvement in the pathophysiology of both motor and sensory symptoms of RLS. These findings emphasize the involvement of pain in RLS and implicate motor symptomatology to occur subsequent to sensory symptoms, thus explaining the effectiveness of opiate medication alongside dopaminergic pharmacotherapy.

## 5. Treatment

Dopaminergic medication remains the first-line treatment option for RLS, with good results in around 70–90% of patients treated with DA agonists. More recently developed non-ergot derived medications (ropinirole and pramipexole) target both D2 and D3 receptors, and cause fewer adverse effects compared to ergot derivatives. A different agent, rotigotine, can be administered through a transdermal patch, enabling sustained DA release. Double-blind randomised-controlled trials have demonstrated oral iron therapy to significantly decrease RLS symptoms severity. Opioid treatment offers a valuable alternative and is the preferred therapy for patients presenting with neuropathy or painful dyskinesia. The anticonvulsant agents pregabalin, gabapentin and its prodrug gabapentin enacarbil have all proven effective in different open-label and double-blind placebo-controlled trials [5].

Treatment should be tailored to the individual patient based on his/her clinical presentation and sensitivity to medication side effects. Iron insufficiency should be promptly corrected with iron supplementation. Pharmacological therapy for RLS can be separated into DA agonists, opioids and antiepileptic medications [10,40].

### 5.1. Dopaminergic agents

Dopaminergic agents have demonstrated high effectiveness in randomized controlled trials, by relieving motor symptoms and improving sleep quality [41,42]. DA agonists (ropinirole, pramipexole, pergolide) are recommended as first-line agents, although frequent adverse effects include nausea, congestion, insomnia, fluid retention and behavioural symptoms, such as hallucinations. The phenomenon of augmentation (symptoms become more severe and widespread, and occur earlier throughout the day) can result from long-term L-dopa and DA agonist intake and should be considered if gradual exacerbation of symptoms occurs subsequent to improvements with pharmacotherapy. As augmentation cannot be avoided by replacing a dopaminergic medication with another dopamine-acting drug, dopaminergic agents should be discontinued and replaced by other medication classes as soon as augmentation is detected. Rebound of RLS can occur during DA therapy withdrawal, however symptoms reduce to their original intensity within a week [10].

### 5.2. Opiates

RLS severity, quality of sleep and night leg activation have all significantly improved with opiate medications as shown in several double-blind placebo-controlled investigations [40]. Augmentation is also absent with use of opioids. Opiates can be a useful treatment option for patients with RLS experiencing pain symptoms or neuropathies; patients who are unresponsive to one codeine-associated medication can still respond to other agents within this pharmacological class [10].

### 5.3. Anticonvulsants

Gabapentin has been shown to improve subjective symptoms, PLMS and sleep quality in a double-blind crossover study and 3 open-label investigations [40]. However increased appetite, retention of fluid, somnolence and dizziness were reported as frequent adverse effects. Lamotrigine has also demonstrated effectiveness in 2 smaller open-label investigations, with adverse effects comparable to GBP. Steven-Johnson syndrome along with allergic skin responses have also been reported with Lamotrigine. It has been suggested that sufferers experiencing painful symptoms or neuropathy should receive anticonvulsants as first-line treatment [10]. Gabapentin Enacarbil (GE), a prodrug of GBP, has recently demonstrated effectiveness in RLS and could be a potentially useful therapeutic option [43].

## 6. Conclusion

Research conducted over the last few years has provided valuable insights into possible pathophysiological mechanisms for RLS. No single underlying cause has been identified, however several hypotheses have been suggested. Three main pathophysiological models have been proposed, namely dopaminergic dysfunction, iron deficiency and opiate abnormalities. There is also evidence for complex interactions between several neurotransmitter systems, including noradrenaline and GABAergic pathways [44,45]. Iron is a co-factor for the enzyme TH necessary for DA synthesis alongside other proteins included in general neuronal activity [44]. Iron deficiency could consequently affect TH functioning and DA production, resulting in the clinical picture of RLS. Not surprisingly, medical conditions associated with reduced iron concentrations increase the likelihood of developing RLS [46]. Furthermore, iron levels demonstrate a circadian rhythms, with 50–60% reductions in the serum concentrations correlating with in symptom exacerbations during the night [47]. Within the basal ganglia, the substantia nigra is seen as a crucial motor system in RLS pathophysiology, with reduced iron absorption and storage [44]. Inter-related iron and DA abnormalities appear to be the main contributors to RLS pathophysiology, as demonstrated by a variety of investigations including imaging, autopsy and animal models. The consistent therapeutic effectiveness from dopaminergic agents and iron supplementation provides further confirmation for this hypothesis. However, interplay could also occur between DA and endogenous opiate pathways. Malfunctioning of these interactions could produce sensory abnormalities within the dorsal horn and behavioural (motor) responses at the level of the spinal pattern generator [44]. The possible interactions between different neurotransmitter systems should guide further neuropharmacological research in order to improve therapeutic efficacy for this relatively common and disabling condition.
